# Immunotherapy reversed myopathy but not cardiomyopathy in a necrotizing autoimmune myopathy patient with positive anti-SRP and MDA-5 autoantibodies

**DOI:** 10.1186/s12872-021-01900-2

**Published:** 2021-02-12

**Authors:** Xue Ma, Li Xu, Yue Li, Bitao Bu

**Affiliations:** Department of Neurology, Tongji Hospital, Tongji Medical College, Huazhong University of Science and Technology, No. 1095, Jiefang Avenue, Qiaokou District, Wuhan, China

**Keywords:** Anti-SRP and anti-MDA5 positive NAM, Cardiomyopathy, Heart transplantation, Autophagy, Apoptosis

## Abstract

**Background:**

Necrotizing autoimmune myopathy (NAM) is pathologically characterized by myofiber necrosis and regeneration with paucity or absence of inflammatory cells in muscle biopsy. Two autoantibodies, namely anti-signal recognition particle (SRP)-antibodies and anti-3-hydroxy-3-methylglutaryl-CoA reductase (HMGCR)-antibodies, are typically specific with NAM. Anti-SRP-positive NAM can be associated with cardiomyopathy which responds well to immunotherapy. Here we reported an anti-SRP-antibody and anti-MDA5-antibody NAM patient who developed severe cardiomyopathy after gaining significant improvement of myopathy and subsequently accepted heart transplantation.

**Case presentation:**

A NAM case with both positive anti-SRP and MDA-5 antibodies who gained significant improvement of the skeletal muscle weakness with immunotherapy, but 3 years later he developed severe dilated cardiomyopathy and at last received heart transplantation. Myocardial biopsy showed disarranged and atrophic myofibers, remarkable interstitial fibrosis without inflammatory infiltrates. Immunohistochemistry analysis revealed increased polyubiquitin-binding protein p62/SQSTM1 protein expression and the positive staining of cleaved-caspase 3 in a few cardiomyocytes. After the transplantation, the patient was symptom-free on oral prednisone (10 mg/day) and tacrolimus (2 mg/day).

**Conclusions:**

We described the first case of anti-SRP and anti-MAD5 positive NAM who had received heart transplantation because of cardiopathy. Though the myopathy had been clinically improved after immunotherapy, the cardiomyopathy remained progressive and lethal. The processes of dysfunctional autophagy and augmented apoptosis were putatively pathophysiological mechanisms underlying cardiac damage in anti-SRP and anti-MAD5 positive NAM.

## Background

Necrotizing autoimmune myopathies (NAM) have been recognized as a subgroup of idiopathic inflammatory myopathies which are characterized by prominently necrotic and regenerating myofibers with no or paucity of inflammatory cells. Currently, NAM is typically associated with the presence of autoantibodies directed against two distinct antigens, namely signal recognition particle (SRP) or 3-hydroxy-3-methylglutaryl-CoA reductase (HMGCR) [1, 2].

SRP, a ribonucleoprotein complex, servers as guiding nascent polypeptides into the rough endoplasmic reticulum [3]. Anti-SRP antibodies firstly were described in polymyositis 30 years ago [4], which recognize antigenic epitopes at the surface of skeletal muscle and trigger complement activation cascade in NAM [5]. Anti-SRP-positive NAM may cause secondary cardiomyopathy [6–10]. However, the underlying mechanism of cardiac damage in anti-SRP positive NAM remains unknow. Anti-melanoma differentiation-associated protein 5 (MDA5) antibodies identified as a dermatomyositis-specific autoantibodies were strongly associated with cutaneous involvement and interstitial lung disease [11]. Limited literature on cardiac damage relevant to anti-MDA5 antibodies were found [12, 13]. Here, we report a NAM case with anti-SRP and anti-MDA5 antibodies who had cardiomyopathy and received heart transplantation though the skeletal myopathy had improved after immunotherapy.

## Case presentation

**Fig. 1:**
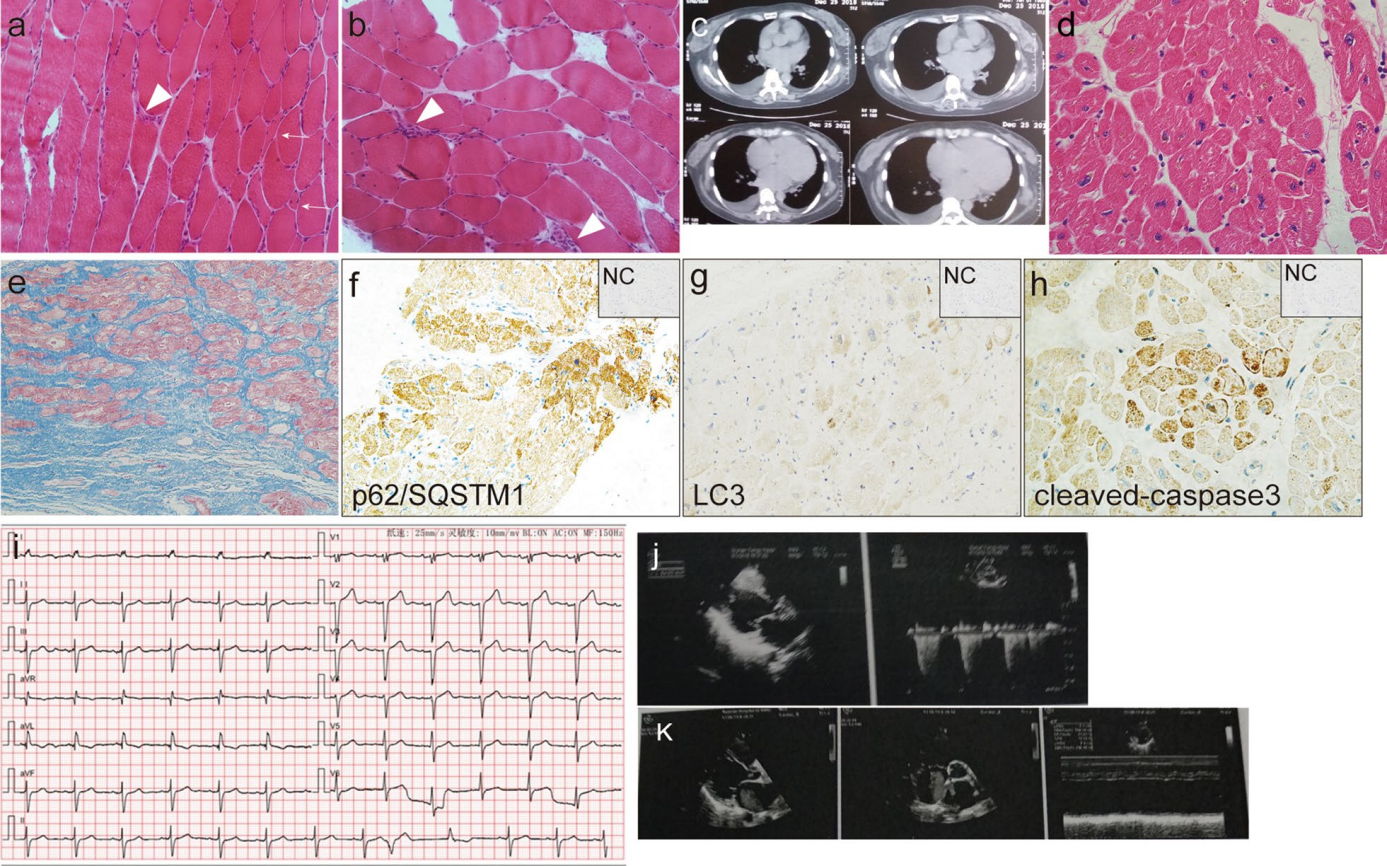
Histopathological findings of the skeletal muscle, cardiac tissue, and chest CT findings. Hematoxylin and eosin staining of the skeletal muscle showed scatter atrophic fibers (white arrow) and necrotizing myofibers (white arrowhead) (**a** × 200; **b** × 200). Enlarged heart was observed on the chest CT (**c**). Hematoxylin and eosin staining (**d** × 400) and Masson staining (**e** × 200) of the left ventricle revealed myofiber disarrangement, atrophy, and interstitial fibrosis with the absence of inflammatory infiltration. Immunohistochemistry analysis displayed p62/SQSTM1 was positive in some cardiomyocytes (**f** × 200). LC3 merely positively stained few cardiomyocytes (**g** × 200) and cleaved-caspase 3 mainly expressed on atrophic cardiac myofibers (**h** × 400) [inset negative control (NC) in **f**–**h**]. ECG demonstrated sinus rhythm, left anterior fascicular block and multifocal ventricular premature beat (**i**). Echo showed dilated left atrium and left ventricle (**j** in 2016 and **k** in 2019)

A 43-year-old Chinese woman, presented with slowly progressive proximal limb weakness for 6 months in 2013. The patient had no family history of cardiomyopathy or sudden cardiac death. Physical examinations showed symmetrically proximal limb weakness (manual muscle testing (MMT) score: 4/5]. Laboratory examinations disclosed increased serum creatine kinase (CK) level (711 U/L; normal range 24–170) and lactic dehydrogenase (LDH) levels (263 U/L; normal range 135–214). Cardiac involvement was documented at elevated troponin levels (0.091 ng/ml; normal range < 0.028). The panel of myositis-associated autoantibodies and myositis-specific autoantibodies screened for this patients included anti-Mi-2α, anti-Mi-2β, anti-TIF1γ, anti-MDA5, anti-NXP2, anti-SAE1, anti-Ku, anti-PM-Scl100, anti-PM-Scl75, anti-Jo-1, anti-SRP, anti-PL-7, anti-PL-12, anti-EJ, anti-OJ, anti-Ro-52, and anti-cN-1A antibodies. In addition, anti-ribonuclear protein, anti-mitochondrial antibodies, anti-dsDNA antibodies and connective tissue disease-related factors were also screened. The determination for anti-SRP antibodies (++), anti-MDA5 antibodies (+), and anti-Ro-52 antibodies (+++) were positive. Electrocardiogram was normal except for frequent ventricular extrasystoles. No abnormalities were detected at echocardiography. Electromyography displayed myogenic damage. The biopsied sample from the right quadriceps revealed scattered myofiber atrophy and necrosis with scarce lymphocytic inflammation (Fig. 1a, b). Thus, the diagnosis of NAM with positive anti-SRP-antibodies and anti-MAD5-antibodies was made, then she was initially treated with oral prednisone at 40 mg/day. Two months later, tacrolimus at 3 mg per day was added because no improvement of the muscle weakness was achieved. The combination therapy of oral prednisone at 10 mg per day and tacrolimus 3 mg per day yielded a good recovery of muscle weakness. Unfortunately, she experienced a severe relapse in 2016 after she discontinued tacrolimus for half a year because she felt normal. She frequently suffered from shortness of breath after exercises since 2016.The limb muscle strength was scored at 3/5 on MMT. Serum CK and LDH levels were elevated to 1875 U/L (normal range 24–170) and 334 U/L (normal range 135–214) respectively. Electrocardiogram showed the left anterior fascicular block and multifocal ventricular premature beats (Fig. 1i). Serum anti-SRP (++), anti-Ro-52 (+++) and anti-MDA5 (+) antibodies remained all positive. Serum N-terminal pro B-type natriuretic peptide (NT-proBNP) and creatine kinase-MB isoenzyme (CK-MB) sharply increased [4526 pg/ml and 15.2 ng/ml respectively; normal < 18.4 (NT-proBNP), normal range 0–5(CKMB)]. Cardiac ultrasonography detected left ventricular enlargement and reduced deceased left ventricular ejection fraction (48%) (Fig. 1j). Computed tomography (CT) of the chest showed bilateral lobar emphysema, heart enlargement, and pulmonary artery enlargement. Oral methotrexate at 10 mg per week in combination with oral prednisone 10 mg per day led to a considerable remission again without noticeable side effects months later.

From 2016 to 2019, her muscle strength almost returned to a normal range, but she occasionally complained of palpitation, chest tightness and shortness of breath. In 2019, the functional class evaluated using 6MWT was approximately 100 m and NYHA class was IV. The serum CK level was normal, yet the NT-proBNP and CK-MB levels remained elevated [up to 11142 pg/ml and 7.46 ng/ml respectively, normal < 18.4(NT-proBNP), normal range 0–5(CKMB)]. Echocardiography showed that significant biventricular enlargement, increased indirect signs of elevated filling pressures (mean E/e ratio 32), a severely reduced LVEF of 25% in absence of significant valvopathies, and an estimated pulmonary artery pressure of 36 mmHg (Fig. 1k). The heart enlargement was evident on chest X-ray and chest CT (Fig. 1c). The patient had no symptoms of myocardial ischemia and coronary angiography was normal. Then the diagnose of dilated cardiomyopathy (end-stage) was made. In May 2019, the patient underwent heart transplantation because of the persistent invaliding symptomatology despite an optimized medical therapy. Currently, tacrolimus at 2 mg a day and 10 mg prednisone a day were employed to prevent transplant rejection and the relapses of myopathy. The symptoms of heart failure have disappeared for months since the transplant operation.

Hematoxylin and eosin staining (Fig. 1d) and Masson staining (Fig. 1e) of biopsied ventricular tissues showed that disarranged myofibrils, atrophic myofibers with remarkable interstitial fibrosis and the absence of inflammatory infiltration. The expression polyubiquitin-binding protein p62/SQSTM1 was observed in many cardiac myofibers (Fig. 1f). LC3, microtubule-associated protein 1A/1B light chain, merely positively stained few cardiomyocytes (Fig. 1g). The immunohistochemistry analysis also showed positive staining of cleaved-caspase 3 in a few atrophic cardiomyocytes (Fig. 1h).

## Discussion and conclusions

The case is the first reported patient with dual positive anti-SRP-antibodies and MDA5-antibodies NAM who developed severe cardiomyopathy after the presentation of NAM had been significantly improved and subsequently accepted heart transplantation. The documented data of the case indicated the myopathy responded well to the combination therapy of oral prednisone and tacrolimus and a severe relapse of myopathy occurred when tacrolimus was discontinued. Retreatment of oral prednisone and methotrexate was able to improve the symptoms and to normalize the CK level. However, a slowly progressive cardiomyopathy was observed 3 years after the onset of NAM, manifested by progressive symptoms of heart failure and changes on ultrasonography, chest X-ray and chest CT. Though the immunotherapy improved the myopathy clinically, the cardiomyopathy remained progressive and lethal. During the whole course, the titers of anti-SRP-antibodies and anti-MDA5-antibodies remained elevated.

Cardiac abnormalities had been observed in patients with anti-SRP-positive NAM [6, 9, 10, 14–17] and previous study had suggested preferential involvement of the cardiac conduction system in anti-SRP-positive patients [14]. Even though anti-MDA5 antibodies were also identified in the patient, there was a lack of correlation between anti-MDA5 antibodies and cardiac involvement [11]. We have found 3 individuals reported on anti-MDA5 positive clinically amyopathic dermatomyositis presenting with severe cardiomyopathy till now [12, 13] and no study on NMA with dual anti-SRP antibodies and anti-MDA5 antibodies involved in cardiac damage. The pathogenic role of anti-Ro52 antibodies in cardiac tissue remains elusive. Earlier research showed anti-Ro52 antibodies did not bind to the surface of cardiac cells [18], yet a recent study suggested anti-Ro52p200 (amino acid 200–239) antibodies induced cardiac electrophysiological abnormalities in children with congenital heart block [19]. There is no study to date on the cardiac involvement in adults with anti-Ro52 antibodies. Thus, the cardiac damage of the case was more likely to attribute to anti-SRP and anti-MDA5 antibodies but not anti-Ro-52 antibodies.

Autophagy mediates the transfer of damaged proteins and intracellular organelles to the lysosomes [20]. Activated autophagolysosomal pathways had been described in NAM biopsy samples [21–23]. Similarly, autophagy markers p62/SQSTM1 accumulation further increased p62/SQSTM1 expression in the cardiac tissue, implying that autophagy dysfunction might play a role in the pathogenesis of cardiac disorders in NAM. Besides, the expression of cleaved-caspase 3 in a few atrophic cardiomyocytes was noted. It is therefore possible that caspase 3-mediated apoptosis contributed to cardiomyocyte apoptosis, myocardial fibrosis, abnormal heart function and eventually, heart failure. Autophagy can suppress apoptosis by inactivation of caspases under certain pathological circumstances, whereas promote apoptosis during other stress conditions [24]. The functional relationship between autophagy and apoptosis needs to be further investigated in cardiac disorders of anti-SRP and anti-MAD5 positive NAM.

Why the autoimmunity in the skeletal muscles responded well to immunotherapy but the cardiac damage was independent and unresponsive simultaneously in this case? There has been two reported cases with anti-SRP antibodies who suffered acute congestive heart failure responded well to prednisone, tacrolimus, and intravenous immunoglobulins or prednisone, plasma exchanges, and rituximab [8, 9]. In this case, the fact that the patient developed chronic heart failure with steroid and immunosuppressants may be due to the more complex mechanisms underlying cardiac damage mediated by autoimmunity. Lines of evidence demonstrated that skeletal muscles are able to regenerate after damage [25, 26]. The recovery of muscle strength of the weakened limbs and the normalization of CK levels should be the result of regeneration after the autoimmunity-mediated necrosis initiated by anti-SRP-antibodies had been significantly suppressed by immunotherapy. In sharp contrast to the ability of skeletal muscles to regenerate, the adult human heart possesses very limited regenerative capacity [27, 28].

This case highlights the cardiac involvement in an anti-SRP and anti-MDA5 positive NAM patient even after the skeletal myopathy has been improved. The autophagy dysfunction, caspase 3-mediated apoptosis, and the lack of ability to regenerate of cardiac myofibers may be important concerns. Further studies need to investigate the pathogenic role of anti-SRP and anti-MDA5 antibodies in secondary cardiac involvement in NAM and to develop targeted treatment.

## Data Availability

All data generated or analyzed during this study are included in this published article.

## Abbreviations

CK: Creatine kinase
CKMB: Creatine kinase-MB isoenzyme
CT: Computed tomography
HMGCR: Hydroxy-3-methylglutaryl-CoA reductase
LDH: Lactic dehydrogenase
MMT: Manual muscle testing
MDA5: Melanoma differentiation-associated protein 5
NAM: Necrotizing autoimmune myopathy
NT-proBNP: N-terminal pro B-type natriuretic peptide
SRP: Signal recognition particle
